# Comparison Between L-312 Hydrophobic-Hydrophilic Acrylate and US-860 UV Hydrophilic Acrylate IOL Opacification Characteristic

**DOI:** 10.3389/fmed.2022.873684

**Published:** 2022-04-08

**Authors:** Jin Xie, Jie Sun, Ting Liu, Shilan Mao, Yunhai Dai

**Affiliations:** ^1^Department of Ophthalmology, Graduate College of Shandong First Medical University, Jinan, China; ^2^State Key Laboratory Cultivation Base, Shandong Provincial Key Laboratory of Ophthalmology, Qingdao, China; ^3^Eye Institute of Shandong First Medical University, Qingdao Eye Hospital of Shandong First Medical University, Qingdao, China; ^4^People's Hospital of Yinan, Linyi, China

**Keywords:** hydrophobic-hydrophilic acrylate IOL, calcium, glistenings, opacification, hydrophilic acrylate IOL

## Abstract

**Objective:**

To compare opacity characteristics of US-860 UV and L-312 IOL, and report the phenomenon of glistenings in hydrophobic-hydrophilic acrylic IOLs.

**Setting:**

Qingdao Eye Hospital.

**Design:**

Experimental study.

**Methods:**

Four medical records (4 eyes) of patients with L-312 or US-860 UV IOL opacification reporting decreased or lost vision who underwent IOL explanation between 2019 and 2021 were reviewed. Explanted IOLs were analyzed by slit-lamp examination, confocal microscopy, scanning electron microscopy (SEM) and energy-dispersive X-ray spectroscopy (EDS) at Qingdao Eye Hospital and Qingdao university of science and technology.

**Results:**

The 4 explanted IOLs were represented by 2 hydrophilic acrylic designs. The preoperative mean corrected distance visual acuity changed from 1.84 ± 1.09 logarithm of the minimum angle of resolution (log MAR) to 0.20 ± 0.03 log MAR postoperatively except case 3. The mean interval of the L-312 IOL was 56.67 ± 14.19 months (range 44 to 72 months), and the interval of the US-860 UV IOL was 27 months. Morphological findings were surface, subsurface calcifications of the US-860 UV IOL material, and the optical region of L-312 IOLs are teeming with a great number of vacuoles by light microscope, scanning electron microscope and Energy Dispersive X-ray Spectral.

**Conclusion:**

The cause of US-860 UV opacification was primary calcification, and vacuoles resulted in L-312 IOL opacification.

## Introduction

With the development of cataract phacoemulsification technology, intraocular lens (IOL) opacification, as one of its complications, had gradually attracted the attention of researchers and clinicians. Cataract is one of the diseases with the highest morbidity, disability, and blindness in the world, which has been a serious threat to the health and quality of life of the middle-aged and elderly people (1). Cataract ultrasonic phacoemulsification and IOL implantation is the most effective method for cataract treatment (2). However, but there are chances of serious complications (such as infective endophthalmitis after cataract surgery, capsular block syndrome, toxic anterior segment syndrome, posterior capsular opacification, IOL opacification, et al.) and irreversible loss of vision associated with the surgery (3). While the occurrence of IOL opacification has declined with the use of modern surgical techniques, recently developed surgical materials and IOL design, IOL opacification can still occur even following uneventful cataract surgery.

With the development of materials science and Chemical Technology, biosynthetic materials are widely used in biology, medical treatment, chemical detection (4). Nanoparticles can retain the biocompatibility of materials and play a role in drug delivery systems (5). Frohn (6) found that premature aging of the ultraviolet blocking agent is one of the reasons for IOL opacification, and some nanocomposites can be used as photocatalysts to affect the stability of compounds (7). Microstructures such as nanoparticles need to be observed and detected by scanning electron microscope (SEM) (8–10), and Energy Dispersive X-ray Spectral (EDS) can analyze the composition of chemical elements (11). In previous studies, SEM and EDS were used to analyze IOL opacification. By comparison with hydrophobic acrylic IOLs, Hydrophilic acrylic IOLs have higher tissue compatibility due to a higher water-content and higher uveal biocompatibility. Since the 1990s, an increasing number of studies report opacification of hydrophilic acrylic IOLs. According to the related research reported that hydrophilic acrylic IOL is more likely to become opacification (12, 13).

In the current study, we describe late postoperative opacification after cataract surgery with implantation of 1 case hydrophilic acrylic and 3 cases hydrophilic-hydrophobic acrylic IOL, and compare the characteristics of two different types of late IOL opacifications. In addition, IOL exchange can improve final visual acuity.

## Methods

This retrospective case series study was performed at the Qingdao Eye Hospital of Shandong First Medical University. This study was approved by the ethics committee of the Qingdao Eye Hospital of Shandong First Medical University (Qingdao, China), and all procedures adhered to the tenets of the Declaration of Helsinki. All patients signed the informed consent of operation and anesthesia before cataract surgery. After the operation, they allowed to use their clinical data and signed written informed consent.

Collecting retrospective data regarding general and ophthalmologic conditions of several patients with postoperative opacification of IOL and a significant visual acuity impairment were referred for IOL replacement to the eye hospital from August 2019 to March 2021, including the patient's condition, date of IOL implantation, IOL serial number, IOL type, intraoperative and postoperative complications, and additional intraocular surgical procedures. All patients had cataract surgery in Qingdao Eye Hospital by two different cataract surgeons. Phacoemulsification was performed under topical anesthesia through a 2.8 mm incision with the Visalis 100 (Alcon Laboratories, Inc.). A balanced salt solution (BSS, Alcon) and healon LG were used in the surgery. The diagnosis of IOL opacification was based on a careful slit lamp examination.

Ophthalmologic evaluation including visual acuity, corrected distance visual acuity (CDVA, Snellen) assessment, ultrasound biomicroscopy (UBM), tonometry, and fundus examination including macular optical coherence tomography, retinoscopy and anterior segment tomography was performed preoperatively and 1 week postoperatively.

All patients accepted povidone iodine rinsed the conjunctival sac before surgery, Intraocular irrigation solution, prepared by 0.75 g Cefuroxime sodium, 5 mg dexamethasone, and 500 ml balance salt solutions, was used during surgery. After operation, the operated eyes received dexamethasone/tobramycin eye ointment. Besides, a combination of tobramycin dexamethasone eyedrops, prednisolone acetate 1.0% and levofloxacin 0.5% eyedrops administered four times a day for 2 weeks and then nonsteroidal antiinflammatory eyedrops were administered four times per day for 4 weeks postoperatively.

The IOL replacement surgery need to save the capsule during mobilization and remove the opacified IOL through a 3.0 mm corneal tunnel, sometimes by cutting the IOL optic into two halves with Vannas/micro scissors, and then, implant a new IOL. The opacified IOLs were sent in the dry state to the Shandong Key Laboratory of Ophthalmology, Shandong Eye Institute, Qingdao, China, where gross and microscopic analyses were performed, and then sent to test in Qingdao University of Science and Technology by SEM coupled with EDS.

In our study, CDVA was measured with the Snellen chart at 5 meters and was then converted to logarithm of the correct minimum angle of resolution (log MAR) values for statistical calculations. Patients who could only perceive hand motion at 2 feet (or less) were considered to have Snellen equivalent 20/20 000 (3.0 log MAR) vision.

The statistical analysis was done with the help of SPSS software version 23.0 (Armonk, NY: IBM Corp.).

## Results

Table 1 shows the general characteristics of each of the 4 cases analyzed so far. The mean patient age at the time of IOL opacification was 71.25 years ± 16.41 (SD) (range 47–83 years). There were three women and one man, and three left eyes and one right eye. All patients with an opacified IOL had cataract surgery in both eyes except case 1 between 2015 and 2017.

**Table 1:** Characteristics of the 4 cases opacity IOLs.

**PT**	**PT 1**	**PT 2**	**PT 3**	**PT 4**
Sex	Female	Female	Male	Female
Age (year)	83	76	47	79
Associated disease	Meningioma, thyroidectomy	Diabetes, hypertension, NPDR	Diabetes	Hypertension, angle closure gla-ucoma
UBM	Normal	Normal	Normal	Normal
Fundus	Normal	Normal	Normal	Normal
IOL	L-312	L-312	L-312	US-860UV
IOP-1 (mmHg)	11	11.1	14	11
IOP-2 (mmHg)	15	9	16	11
HbA1c (%)	-	-	8.6	5.8
Eye-1	OD	OU	OU	OU
Eye-2	OD	OS	OS	OS
IT (month)	72	55	44	27
SN	91308761001	91307941002	91310521013	O-04817001-005
IOL model	AR40e	AR40e	iSert 251	SoftecHD
Visual acuity-1	0.15	0.001	1.0	0.02
CDVA-1	0.15	0.001	1.0	0.02
Visual acuity-2	0.15	0.6	1.0	0.7
CDVA-2	0.6	0.6	1.0	0.7

*PT, patient; HbA1c, hemoglobin A1c; IT, interval time; SN, serial number; UBM, ultrasound biomicroscopy; IOP1, IOP before IOL replacement; IOP2, IOP after IOL replacement; Eye-1, eyes of cataract surgery; Eye-2, eyes of IOL opacification; Visual acuity-1, Visual acuity before IOL replacement; Visual acuity-2, Visual acuity after IOL replacement; CDVA-1, CDVA before IOL replacement; CDVA-2, CDVA after IOL replacement; NPDR, nonproliferative diabetic retinopathy; “-”, express no accurate information*.

Case 1 has a history of coronary insufficiency and underwent meningioma and thyroidectomy before 10 and 20 years ago, and case 4, diagnosed with high blood pressure, underwent gonioplasty in left eye because of angle closure glaucoma (PACG) during the cataract surgery. Patient two and patient three were diagnosed a diabetic. In addition, patient two had nonproliferative diabetic retinopathy (DR) and hypertension.

Intraocular pressure before and after the procedure was <21 in all affected eyes, and all the results of UBM, and fundus examination were normal (Table 1). The results of UBM showed that the anterior chamber angles were open, the position of IOLs was acceptable, the retina attached and the lens zonule remained intact, and no problems of macular edema macular scar, and fundus hemorrhage by fundus examination.

**Figure 1:**
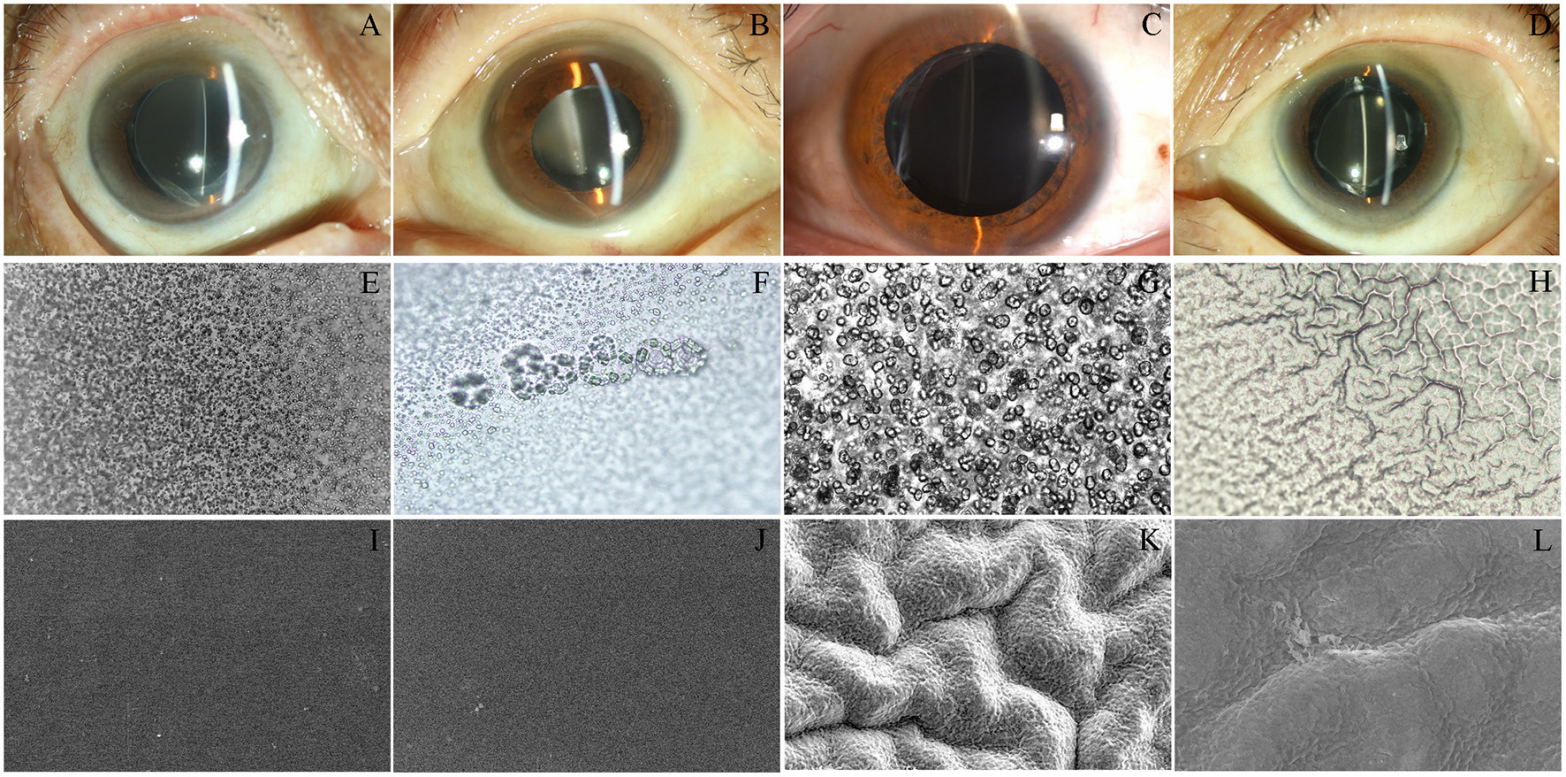
Characteristics of the implanted IOL. Slit-lamp photographs **(A–D)** from the four patients, showing grayish white with a diffuse ground glass appearance on the anterior surface of the lens. **(A–D)** Obtained from case 1, 2, 3, 4. L-312 IOLs **(E–G)** showed a great quantity of vacuoles and US-860 UV **(H)** presented cerebriform under light microscopy (original magnification × 40). **(E–H)** Obtained from case 1, 2, 3, 4. Scanning electron micrographs obtained from case 1 **(I,J)** and case 4 **(K,L)** showed that the surface of L-312 IOL was smooth and US-860 UV IOL appeared as rough mountain. **(I,K)** Original magnification 500, **(J,L)** Original magnification 1,000.

Four opacity IOLs contain two different designs, including 3 cases L-312 hydrophilic-hydrophobic acrylic IOL, and 1 case US-860 UV hydrophilic acrylic IOL. All the IOLs showed a grayish white or brownish opacification with a diffuse ground glass appearance in Slit lamp (Figure 1), and IOL opacification mainly concentrated in the central area of IOL optical part.

In our study, the mean interval of the L-312 IOL between the initial cataract surgery and the IOL exchange because of opacification was 56.67 ± 14.19 months (range 44–72 months), and the time interval of the US-860 UV IOL was 27 months. There were no serious intraoperative complications during IOL replacement surgery, and all IOL implanted into capsular.

The serial numbers of the L-312 hydrophilic-hydrophobic acrylic IOL were 9130871001(IOL#1), 9130791002(IOL#2), and 91310521013(IOL#3), The serial numbers of the US-860UV IOL was O-04817001-005(IOL#4). All replacement IOLs were hydrophobic IOLs, including 2 AR40e IOLs (Abbott Medical Optics, Inc., US), 1 iSert 251 IOL (Hoya Corporation, Japan), and 1 SoftecHD IOL (Lenstec, Inc., US).

The CDVA was equal to visual acuity, therefore, the CDVA was used for comparison in subsequent studies. The CDVA of Case 1, 2, 4 was a significant improvement after IOL exchange (mean CDVA 1.84 ± 1.09 log MAR to mean CDVA 0.20 ± 0.03 log MAR) compared with case 3. Regarding this patient, 47 years old, although the degree of opacification of the L-312 IOL relatively light, and had no obvious influence upon the vision, but a large number of glistenings caused a significant straylight increase and glare-related problems, and had a significant impact on patients' life (especially night driving). All patients recovered well postoperatively, with no complications. Therefore, IOL replacement can safely and effectively improve the visual acuity and visual quality of patients.

Light microscope images of the explanted IOLs from Case 1,2,3 show a characteristic vacuole-like structure and surface lesions extending into the body of the L-312 IOL, but the analysis of the explanted IOL from Case 4 showed cerebriform and granular appearance of the surface of the cloudy US-860 UV IOL (Figure 1). The characteristic of L-312 IOL opacification were markedly different to those in Case 4.

**Figure 2:**
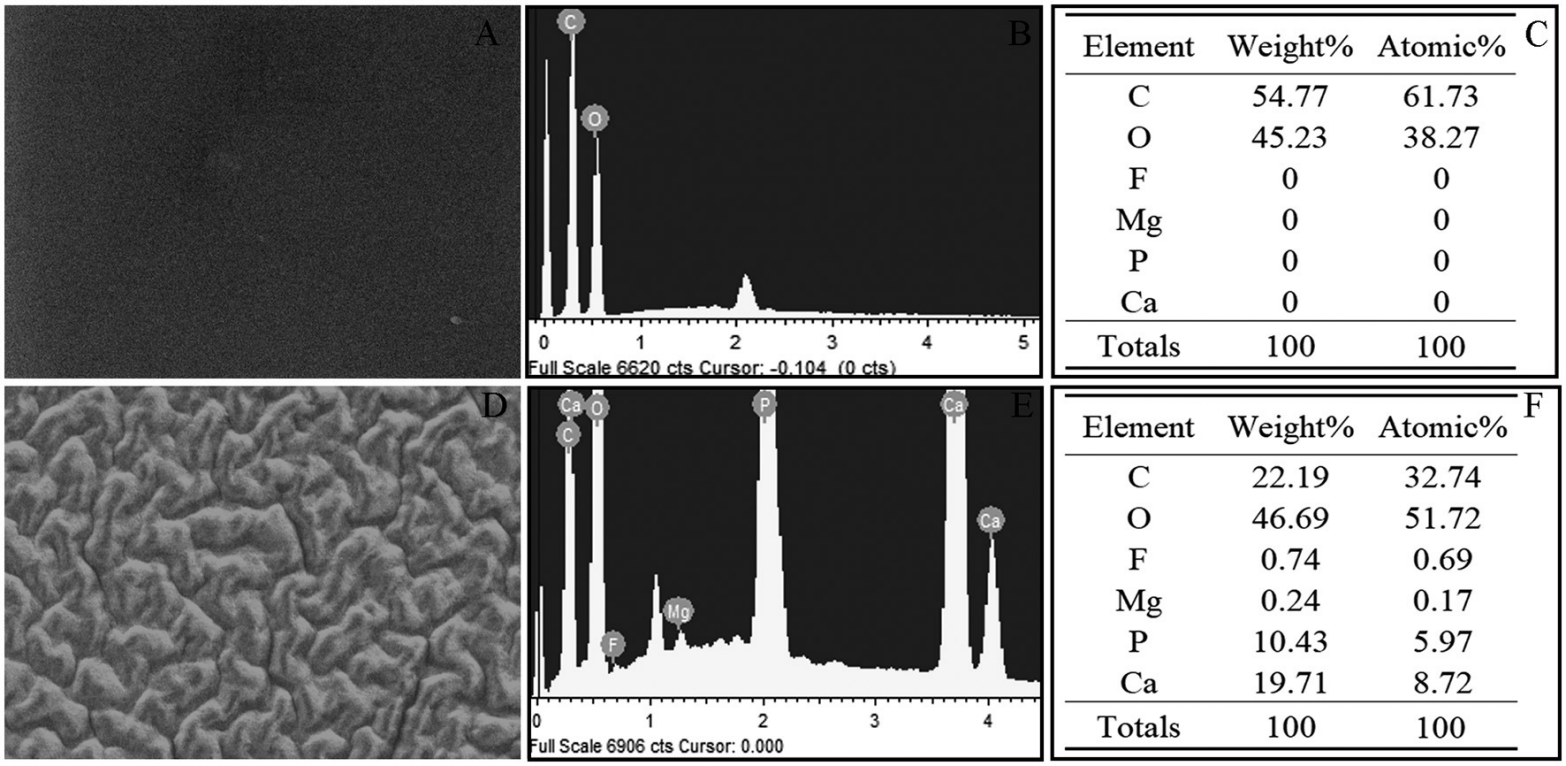
Energy Dispersive X-ray Spectral from case 4 revealed the presence of calcium and phosphorous in the turbid area of US-860 UV, but L-312 IOL (case 1) contains only carbon and oxygen. **(A–C)** Obtained from case 1, **(D–F)** obtained from case 4. C, carbon; O, oxygen; P, phosphorus; Ca, calcium; F, fluorine; and Mg, magnesium.

For case 4, the surface changes that appeared cerebriform by light microscopy appeared as a large number of particles settle on the surface of the crystal to form a gully by electron microscopy. However, the surface of L-312 IOL (IOL#1) showed smooth and clean under SEM, and vacuole-like structure cannot be detected by SEM (Figure 1). Elemental analysis used with EDS (Figure 2) confirmed that the deposits on the US-860 UV IOL surface were composed of calcification and phosphorus, both of which are characteristically absent in normal IOLs of this type. The weight ratio of calcium to phosphorus in opacity area of IOL was about 2:1, and the atomic ratio was about 3:2. Besides, trace amounts of magnesium (0.24%) and fluorine (0.74%) were also found. Nevertheless, there were no other elements except carbon and oxygen in opacity L-312 IOL (IOL#1). The results showed that the turbidity types of the two different intraocular lenses were different.

## Discussion

In recent years, with the increasing reports of long-term postoperative IOL opacity, it has attracted the attention of ophthalmologists. The types of IOL mainly includes silicone IOL, Poly Methyl Methacrylate (PMMA) IOL, hydrophobic acrylic IOL and hydrophilic acrylic IOL. IOL opacifications has been reported in all types of IOL, mainly including calcification, glistening, subsurface nanoglistenings and snowflake degeneration (14, 15). The risk of IOL opacification including severe reduction of visual function, straylight, glare, interfere fundus examination and treatment.

In previous research reports, Calcium and phosphorus deposition was the important common reason for the turbidity of hydrophilic IOL and hydrophobic-hydrophilic acrylate IOL (16–20). Neuhann et al. described three forms of hydrophobic acrylate IOL calcification (21, 22). The first form was called primary calcification, which refers to calcification that is inherent in the IOL, and may be caused by fabrication of the IOL, issues with its packaging process, or inadequate formulation of the polymer. The calcification presumably occurs in otherwise normal eyes and generally is not associated with comorbidities. The second form was secondary calcification that refers to secondary superficial calcium deposits on the IOL, most likely because of environmental circumstances with changes in the intraocular aqueous milieu. These patients generally have systemic and (or) ophthalmic disease, including diabetes, uveitis, or had vitreoretinal surgery, which can result in fluid–gas exchange and disrupt the blood–aqueous barrier. Therefore, the secondary calcification is not related to any problem with the IOL itself. The third form called false-positive calcification. By definition, the type represents the phenomena when another pathology is mistaken for calcification or false positive staining for calcium occurs.

In this study, we used light microscopy, electron microscopic, as well as elemental or molecular surface analytical techniques by SEM have demonstrated that the opacification was related to calcium and phosphate precipitation on the surface and subsurface of the US-860 UV IOL, and confirmed the previous experimental results. Even if there was no definite evidence that US-860UV IOL opacification was related to its production, package or transportation, but we still considered that US-860UV IOL belong to primary calcification, because we've ensured that the patient (#4) did not experience disease or surgery that resulted in impaired blood–aqueous barrier. It's reported that the most frequently primary calcification IOL designs contained the SC60B-OUV IOL (Medical Development Research, Inc.), and the LS-502-1 IOL (Lentis, Oculentis GmbH) (17, 20, 23). The turbidity characteristics of these two types of IOLs are similar to those of US-860UV IOL.

Hydrophobic-hydrophilic acrylate IOL with a layer of hydrophobic material on the framework of hydrophilic material, so it has the common advantages of the two materials. Paula et al. (10) described a significant number of cases of severe opacification of hydrophobic-hydrophilic acrylic IOLs (LS-502-1 IOL), and all IOLs had a similar pattern of opacification, with yellowish diffuse opacification uniformly distributed and calcium deposits on the surface and/or subsurface of the optic and haptics and within the IOL material. Nicolas (20) considered Lentis LS-502-1 IOL opacification attributed to primary calcification, and might be caused by the interaction of patients' individual factors (altering intraocular ion concentrations), IOL material traits, and manufacturing associated contamination. In 2018 (18), studies of cases involving L-312 IOLs with uneven distributed part calcification were published, and the researchers confirmed a manufacture issue might be the reason for the opacification.

Interestingly, in our study, the L-312 IOL opacification do not seem to be bound up with calcium and phosphate depositions, but more similar to the characteristic of hydrophobic acrylic IOL. Several test results of L-312 IOL suggest that the cause of opacification may be related to glistening.

Glistening has been reported in almost all IOL materials and designs; Nonetheless, it is most common and has the greatest severity in hydrophobic acrylic IOLs (24). It was reported that the risk factors for development of glistening IOL including material properties (water content), time from IOL implantation, temperature changes, breakdown of the blood-retinal barrier (e.g., postoperative inflammation, diabetes, uveitis), glaucoma, and issues related with the IOL packaging process (25, 26).

In 1996, Dhaliwal first reported glistenings of Acrysof (Alcon, Inc., Fort Worth, Texas) hydrophobic acrylic IOLs, and considered the glistenings are likely caused by water vacuoles that form within the lens after hydration within the eyes (27). Wang et al. reported that the change in the temperature of the surrounding environment might be the cause for this vacuolation of IOL by vitro studies (13). Grzegorz et al. immersed the IOLs in a balanced salt solution at temperatures ranging from 37°C to 60°C and cooled to room temperature, and the IOLs created different numbers of glistenings (28). Kang described optic opacification of the IOL (Acrysof® MA60BM Alcon, Fort Worth, Texas, USA) extracted from a 55-year-old male who underwent binocular cataract 11 years ago, and the extracted IOL optics at 4°C, room temperature, and 37°C were transparent at dry conditions. On the contrary, when the dried IOL was immersed in normal saline at room temperature and 37°C, opacification appeared. However, when the dried IOL was immersed in normal saline at 4°C, opacification of the IOL did not appear. They believed that the IOL opacification can occur depending on temperature and hydration conditions (29). In addition, the changes in water content of the IOLs can significantly affect the formation of glistenings, recent studies have indicated that a low water content of hydrophobic acrylic materials (typically <0.5% water) might be partially responsible for glistenings (30). In our present study, because IOL opacification occurred mean 56.67 months after surgery, we therefore believe that hydration resulted in L-312 IOL opacification rather than temperature.

Generally speaking, the US-860 UV hydrophilic acrylic IOL opacification caused by calcification can result in visual impairment so obvious that the patient needs to exchange the opacity IOL; however, the glistenings of hydrophobic acrylic lenses are actually refractile micro vacuoles in the IOL optic formed in aqueous environment, but rarely resulting in blurred vision (31). Meanwhile, the impact of glistenings on postoperative visual function and the evolution of glistenings years after surgery remain controversial, and IOL replacement induced by glistenings has rarely been reported in the current researches (25). Nevertheless, in this study, three patients underwent IOL replacement surgery because of visual impairment caused by calcium and phosphorus deposition or glistenings of IOL, and one patient changed IOL due to the effect of straight light on visual quality.

Combined with previous report (13), the visually significant late postoperative opacification of US-860 UV hydrophilic acrylic IOLs occurred about 12–48 months (mean 24.57 ± 11.40) after the IOL implantation, and all the L-312 IOLs explanted at our center opacified in a various range of time, from 44 to 72 months (mean 57.40 ± 10.09) postoperatively. Paula (17) reported that the mean interval between implantation of the Lentis LS-502-1 hydrophilic–hydrophobic acrylic IOLs and the diagnosis of calcified opacification was 29.15 ± 9.57 months. This interval of glistenings of L-312 IOL is much longer than the interval of US-860 UV IOLs. What's more, although the L-312 IOL (IOL#3) looked obvious turbidity, but the visual acuity was not affected. Therefore, the hydrophobic coatings, attached to the hydrophilic acrylic IOL surface had the function of delaying or preventing IOL opacity and maintaining IOL performance.

According to the time of postoperative IOL opacification, some researchers divided it into early and late postoperative opacification, both L-312 IOL and US-860 UV IOL opacity were late postoperative opacification (32).

The main limitation of this study is the small sample size. More cases, research and long-term follow-ups are necessary to investigate the true influence of individual factors and to confirm whether IOL calcification can be definitely linked to certain circumstances of patient or whether IOL calcification is the unique result of slight differences between every single IOL due to production. Besides, the cause of the glistenings of L-312 hydrophobic-hydrophilic acrylic IOLs is also worth studying further.

In summary, we compared opacity characteristics of US-860 UV and L-312 IOL, and found that the causes and mechanisms of opacification were different. This study expanded the IOL type of IOL opacification, and the study is the first, to our knowledge, reported that L-312 IOL opacification is characterized glistenings. Besides, IOL replacement can safely and effectively improve the visual acuity of patients. US-860 UV IOL opacification was related to calcium and phosphorus deposited on the surface of the IOL optical region because of hydrophilic acrylate material or production, package and transportation of those IOL, but L-312 IOL opacification, called glistenings, caused by a large number of vacuoles, which may be depend on temperature and hydration conditions. In addition, the interval of L-312 hydrophobic-hydrophilic acrylic IOLs was longer than that of US-860UV hydrophilic acrylic IOL in this study, the hydrophobic coating may play a protective role against IOL opacification. We hope with this study, we reveal the detailed features of this phenomenon for future studies to reference and compare, and help assist clinicians in selecting appropriate IOL.

## Data Availability Statement

The original contributions presented in the study are included in the article/supplementary material, further inquiries can be directed to the corresponding author/s.

## Ethics Statement

Written informed consent was obtained from the individual(s) for the publication of any potentially identifiable images or data included in this article.

## Author Contributions

JX, JS, and SM collected and analyzed the data. JX and JS wrote the manuscript. YD, TL, and JX designed the research. All authors contributed to the article and approved the submitted version.

## Funding

This study was supported by grants from the National Natural Science Foundation of the People's Republic of China (81600721) and Qilu Health Outstanding Young Talents Program (A0241).

## Conflict of Interest

The authors declare that the research was conducted in the absence of any commercial or financial relationships that could be construed as a potential conflict of interest.

## Publisher's Note

All claims expressed in this article are solely those of the authors and do not necessarily represent those of their affiliated organizations, or those of the publisher, the editors and the reviewers. Any product that may be evaluated in this article, or claim that may be made by its manufacturer, is not guaranteed or endorsed by the publisher.
